# Electrophysiological Correlates of Character Transposition in the Left and Right Visual Fields

**DOI:** 10.3389/fpsyg.2021.684849

**Published:** 2021-08-04

**Authors:** Er-Hu Zhang, Xue-Xian Lai, Defeng Li, Victoria Lai Cheng Lei, Yiqiang Chen, Hong-Wen Cao

**Affiliations:** ^1^Research Center for Language, Cognition and Language Application, Chongqing University, Chongqing, China; ^2^Centre for Studies of Translation, Interpreting and Cognition, University of Macau, Macau, China

**Keywords:** character transposition, left and right visual fields, N250, N400, LPC

## Abstract

This study examined the brain activity elicited by the hemispheric asymmetries and morpheme transposition of two-character Chinese words (canonical and transposed word) and pseudowords using event-related potentials (ERPs) with a dual-target rapid serial visual presentation (RSVP) task. Electrophysiological results showed facilitation effects for canonical words with centrally presented visual field (CVF) and right visual field (RVF) presentations but not with left visual field (LVF) presentations, as reflected by less negative N400 amplitudes. Moreover, more positive late positive component (LPC) amplitudes were observed for both canonical words and transposed words irrespective of the visual fields. More importantly, transposed words elicited a more negative N400 amplitude and a less positive LPC amplitude compared with the amplitudes elicited by canonical words for CVF and RVF presentations. For LVF presentations, transposed words elicited a less negative N250 amplitude compared with canonical words, and there was no significant difference between canonical words and transposed words in the N400 effect. Taken together, we concluded that character transposition facilitated the mapping of whole-word orthographic representation to semantic information in the LVF, as reflected by the N250 component, and such morpheme transposition influenced whole-word semantic processing in CVF and RVF presentations, as reflected by N400 and LPC components.

## Introduction

The human reading system is a fast, automatic, and comparably robust system, but it is also influenced by factors such as letter space, relative position, and lexical organization (Grainger and Whitney, 2004; Rayner et al., 2006). Although skilled readers could easily understand text with letter transpositions (Davis, 2003), there was a transposition cost during reading, as reflected by longer reading times or lower identification accuracies (Rayner et al., 2006). Moreover, significant transposed-letter similarity effects were found independent of the morphological processing of the letter transposition (Beyersmann et al., 2012). Event-related potential (ERP) studies revealed that transposed-letter non-word pairs (e.g., jugde-judge) could produce a lower N250 effect compared with the substituted-letter control condition (e.g., jupte-judge), but no differences were observed between two word-word priming conditions (e.g., “casual-causal” vs. “carnal-causal”) (Dunabeitia et al., 2009). Notably, the model proposed by Grainger and Holcomb (2009) suggested that the mapping of whole-word orthographic representation to semantic information was related to the N250 component. Additionally, the N400 amplitudes in a lexical decision task varied as a function of stimulus types (real words vs. transposed-letter non-words vs. replacement-letter non-words). Specifically, the N400 effect elicited by transposed-letter non-words (e.g., relovution) was reduced compared with that of replacement-letter non-words (e.g., retosution) (Carreiras et al., 2007). These findings suggested that non-words created by transposing letters were very effective in activating the form-level and semantic-level representation of their original word regardless of letter transposition. Beyersmann et al. (2012) provided further evidence for both whole-word access and morphological decomposition at the initial stages of visual word recognition.

Analogous to English, Chinese readers can identify words with character transpositions in rapid succession; however, a transposition cost is involved in identifying transposed words (e.g., “
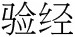
” can be turned to the canonical word “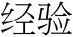,” meaning “experience”) compared with canonical words. Behavioral studies demonstrated that character order of Chinese compound words was not strictly encoded at the early processing stage in Chinese reading (Gu et al., 2015; Cao et al., 2016b), and that readers tended to process two-character Chinese words *via* a holistic approach in children (Liu et al., 2010, 2014). ERP studies demonstrated that transposed words elicited a more negative N400 amplitude than canonical words (Bai et al., 2011; Tong et al., 2014). These results suggested that readers could automatically and effectively retrieve the word semantics of the reversed configuration word even though a detectable influence on the semantic processing of compound word entities was observed and further demonstrated the character positional sensitivity in Chinese reversible word processing. It is notable that these studies primarily explored simultaneously presented whole words rather than sequentially presented characters in visual fields.

Moreover, reading is a complex process involving temporal and spatial information extraction. While reading, the eye movement shifts within a small area of high acuity across the text to assess the identity and the position of the letters in a word available within the visual fields (Potter, 1984; Inhoff, 1990; Besner and Humphreys, 1991; Grainger, 2008; Davis, 2010). Previous studies have demonstrated the right visual field (RVF) advantage in alphabetic and non-alphabetic script reading (Inhoff and Liu, 1998; Rayner, 1998; Darker and Jordan, 2004; Eviatar and Ibrahim, 2004; Willemin et al., 2016; Bergerbest et al., 2017). Such RVF advantage was substantiated by the split fovea claim (Brysbaert, 2004; Hsiao et al., 2007) that the left cerebral hemisphere dominated visual word processing (Gold and Rastle, 2007; Cai et al., 2008), which suggested that the two hemispheres were functionally differentiated in normal reading. The sequential encoding regulated by inputs to oscillations within letter units (SERIOL) model and the split fovea model have made specific predictions regarding letter position encoding in the left and right hemispheres (LH and RH, Whitney, 2001; Shillcock and Monaghan, 2004). The former model claimed that letter position encoding within words was similar for the left and right hemispheres, while the lateral inhibition among adjacent letters would be much stronger in the right hemisphere than in the left hemisphere (Whitney, 2001; Whitney and Lavidor, 2004). In addition, Monaghan et al. (2004) posited that semantic priming effects are stronger in the left compared with the right input of the model. According to the latter model, individual letters were coded in the left hemisphere, whereas a coarser coding was present in the right hemisphere (Monaghan et al., 2004). Shillcock and Monaghan (2004) argued that position-specific encoding in the left hemisphere was more sensitive to transpositions.

Researchers have devoted considerable effort to explore the cognitive mechanisms and electrophysiological correlates of hemispheric language processing by combining the visual half-field presentation technique with the recording of event-related potentials (ERPs) (Atchley and Kwasny, 2003; Deacon et al., 2004; Grose-Fifer and Deacon, 2004; Coulson et al., 2005; Bouaffre and Faita-Ainseba, 2007; Wlotko and Federmeier, 2007, 2013; Metusalem et al., 2016). For alphabetic language studies, some researchers have used lexically associated (e.g., spare-tire) or unassociated (e.g., spare-pencil) word pairs as the prime and target separately, with the prime presented in the center and target presented in the left visual field (LVF) (right hemisphere, RH) or RVF (left hemisphere, LH). The results showed that the associative priming effect appeared through the P200, N400, and late positive component (LPC) time windows. More importantly, the N400 associative priming effect was slightly more significant for RVF than for LVF presentations (Coulson et al., 2005), suggesting a left-right asymmetry. For logographic language investigations, a previous study investigated the behavioral characteristics of character transposition processing of Chinese compound words (canonical and transposed words) and pseudowords in the left and right visual fields using a dual-target rapid serial visual presentation (RSVP) paradigm. The accuracies of canonical words were higher for RVF than for LVF, while the accuracies were almost identical for transposed words in the LVF and RVF. Although the RVF advantage was confirmed for Chinese canonical words, this superiority was modulated by the character transposition processing of compound words (Cao et al., 2016b).

Notably, behavioral indicators only provide explicit measures after meaning access, and issues related to the electrophysiological correlates of character transposition of two-character Chinese word processing among different visual field presentations remain underspecified. RSVP is an effective means of studying the time course of language processing and reading (Potter, 1984). Typically, targets in a rapid stream of successive visual events are presented at the same location in a dual-target RSVP task. The variant of the visual-field technique, in which the targets are spatially shifted to the left or right visual field, has been proven to be an effective means by which to explore hemispheric asymmetries (Hollander et al., 2005; Barber et al., 2011; Asanowicz et al., 2017; Bergerbest et al., 2017). Moreover, as ERPs provide a continuous measure of processing and allocation of attention to stimuli (Luck et al., 2000), they can be very useful for analyzing the time course of attention allocation to a visual detection task. Following the prior behavioral study on horizontal visual fields by Cao et al. (2016a), this study aimed to further examine the brain activity elicited by the hemispheric asymmetries and morpheme transposition of two-character Chinese words (canonical and transposed words) and pseudowords using the ERP technique with a dual-target RSVP task. In each trial, the first target character (T1) and distractor numbers were always presented at the center of the screen (0° eccentricity), and the second target character (T2) was randomly presented either at the center of the screen (0°, centrally presented visual field, CVF), 2° to the left of center (LVF) or 2° to the right of center (RVF). Such manipulation experiments were designed to explore whether canonical and transposed words yield similar or different brain-level responses in the LVF, CVF, and RVF. We hypothesize that transposed words will effectively activate the form and semantic-level representations of whole word entities for CVF or RVF presentations, but that the character transposition will result in a disruption to the form and semantic-level information extraction for LVF presentations, as reflected by amplitude differences in N250, N400, and LPC components.

## Method

### Participants

Thirty-four students participated in the experiments (18–24 years old, mean age = 19.97 years, SD = 1.66 years, 16 males). Three subjects were excluded because of excessive noise in their EEG data. All the participants were right-handed native Chinese speakers and had normal or corrected-to-normal visual acuity with no psychiatric or neurological history. They provided written informed consent before participation, following the ethics protocol of the Institutional Research Ethics Committee of Chongqing University.

### Stimuli

Three categories of two-character word pairs were selected as target stimuli (the two characters were labeled T1 and T2, respectively): (1) 180 canonical words {e.g., “
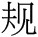
” (T1) and “
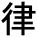
” (T2), in which the two characters can be integrated into a meaningful word in Chinese when they are written together in sequence [“
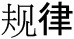
” (T1 + T2), meaning “regulation”]}; (2) 180 transposed words {e.g., “
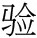
” (T1) and “
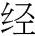
” (T2), in which the two characters cannot form a meaningful word in the order of “
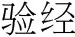
” (T1 + T2) but can form a meaningful word in the reverse sequence [“
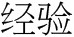
” (T2 + T1), meaning “experience”]}; and (3) 180 two-character pseudowords [the pseudowords are composed of two single-character words, but the combination is meaningless regardless of the order of its constituent characters, e.g., “
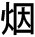
” (T1), meaning “smoke,” and “
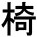
” (T2), meaning “chair”].

The stimuli are very frequently used words and characters selected from the SUBTLEX-CH-WF database (Cai and Byrsbert, 2010) with occurrence frequencies per million provided. Ten postgraduate students who did not participate in the formal experiment were asked to rate the concreteness of each word on a 5-point scale (1 for highly abstract, 5 for highly concrete). The frequencies, word concreteness, and number of strokes were counterbalanced across the stimulus types. Paired-sample *t*-tests showed no significant differences in the frequency and number of strokes between T1 and T2 among the three word types (all *ps* > 0.05). Furthermore, there were no significant differences in the frequency and concreteness between canonical words and the corresponding canonical words of transposed words (both *p* > 0.05). Table 1 illustrates the descriptive statistics of the frequency, concreteness, and number of strokes of the three stimulus types and their characters.

**Table 1:** Descriptive statistics (mean and SD) of the frequency, word concreteness, and number of strokes of the three stimulus types and their characters.

**Stimulus type**		**Frequency**	**Word**	**Number of**
			**concreteness**	**strokes**
Canonical word	T1	49.66 (121.63)		9.80 (2.32)
	T2	40.71 (84.86)		9.89 (2.35)
	T1 + T2	35.81 (86.13)	2.89 (1.01)	
Transposed word	T1	39.30 (70.64)		10.05 (2.41)
	T2	52.15 (121.11)		10.08 (2.59)
	T2+T1	31.46 (50.10)	2.97 (0.93)	
Pseudoword	T1	42.27 (98.36)		9.83 (2.21)
	T2	73.91 (198.31)		9.81 (2.13)

### Procedure

**Figure 1:**
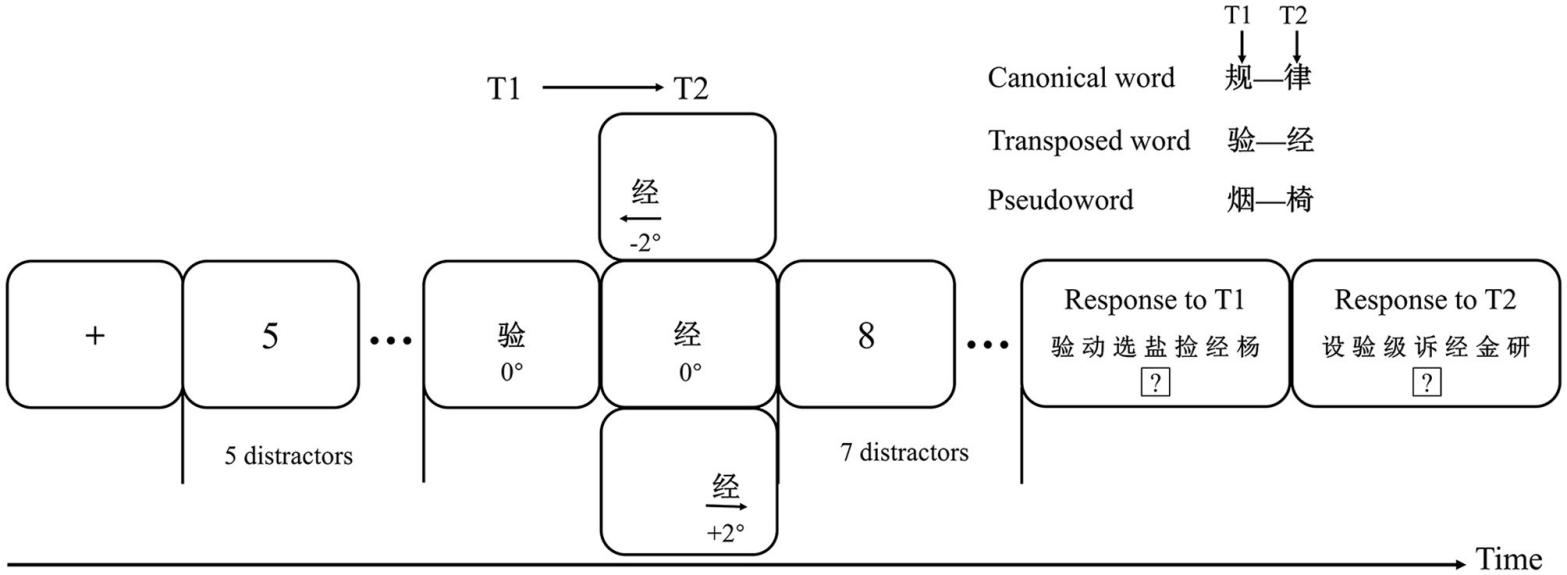
Schematic depiction of the stimuli presentations used in the present experiment. The T1 and T2 combination randomly formed a canonical word, transposed word, or pseudoword, with T1 presented at the center (0°) and T2 presented randomly either at the center (0°, CVF), 2° to the left or right of center (LVF, RVF). The depicted trial is one in which T1 (“
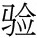
”) and T2 (“
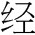
”) could form a meaningless transposed word in the order of “T1 + T2” but could form a meaningful word in the reverse sequence [“
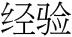
” (T2 + T1), meaning “experience”].

The experiment took place in a soundproof room. Targets were presented in black Courier New font with a font size of 30 against a white background *via* Eprime 3.0 (Psychology Software Tools, Pittsburgh, PA, United States). The participants viewed the stimuli at a distance of 57 cm from the screen so that 1 cm corresponded to an ~1° visual angle. The schematic depiction is shown in Figure 1. Each participant was explicitly instructed to maintain fixation at the center of the screen throughout each trial. Each trial began with a black cross fixation (“**+**”), presented for 1,000 ms. After this, an RSVP stream of five numbers (distractors, from 2 to 9) was randomly presented. Then, a Chinese character (T1) and another Chinese character (T2) appeared sequentially, with T1 presented at the center of the screen and T2 presented randomly either at the center of the screen (0°, CVF), 2° to the left of center (LVF), or 2° to the right of center (RVF). T1 + T2 could be combined into a canonical word, transposed word, or pseudoword with the condition that each word or character was presented only once to avoid repetition effects. Thus, the research design consists of two within-participant independent variables: stimulus type (canonical word vs. transposed word vs. pseudoword), and visual field (LVF vs. CVF vs. RVF). Finally, seven numbers (distractors) appeared after T2. Each item, including distractors, T1, and T2, was presented for 100 ms. After rapid succession, the T1 and T2 response panels appeared in order. The T1 response panel containing T1, T2, and another five new black Chinese characters was presented horizontally in the middle of the screen. The participants were asked to respond to T1 by clicking the mouse on it. Once T1 was chosen, the T2 panel automatically appeared for the participants to identify T2. Each panel lasted for 6 s at most. Before the experiment, the participants were instructed to identify the targets in order and click the “?” below the Chinese characters when they failed to identify the characters. If they clicked the “?,” the next trial was initiated. The participants were asked to avoid eye blinks during the rapid presentation phase.

Each participant began with 10 practice trials to understand the experimental flow. There are 540 trials in total and 60 trials for each condition in the formal experiment section. The participants took a break after every 90 trials.

### EEG Recording and Processing

Electroencephalogram (EEG) data were recorded based on the International 10–20 system with an actiChamp amplifier and 64 active electrodes (Brain Products, Gilching, Germany). The impedance of each electrode was kept below 5 KΩ. The ground electrode was Fpz, and a vertical electrooculogram (VEOG) electrode was pasted 1 cm above the left eye. Data were sampled at 1,000 Hz, and the online frequency range of the amplifier was 0.01–70 Hz with Cz as the original reference.

Offline analysis was performed using BrainVision Analyzer 2.1. The reference was transformed to the left and right mastoid sites (i.e., TP9 and TP10). The band-pass filter was set as 0.5–30 Hz with a slope of 24 dB/oct and a notch of 50 Hz. Independent component analysis (ICA) was performed for electrooculogram (EOG) correction. Semiautomatic data inspection was set to reject the artifacts with the criteria of a maximally allowed voltage step of 50 μV/ms, a maximal allowed absolute difference of 200 μV, and a lowest allowed activity level of 0.5 μV. The EEG data were segmented based on T1 only when both T1 and T2 were correctly identified in order, beginning at the baseline 200 ms before the T1 onset and lasting 900 ms. Baseline correction was performed during the 200-ms pre-T1 onset period. All EEG segments with amplitudes beyond ±80 μV were rejected as artifacts. In the final analyses, the average number of artifact-free trials for each participant in the nine conditions was 48 (SD = 6.8), 45 (SD = 9.1), 44 (SD = 6), 51 (SD = 5), 49 (SD = 6), 48 (SD = 7.3), 51 (SD = 5.1), 50 (SD = 6.2), and 50 (SD = 5.9), which guaranteed a good signal-to-noise ratio.

### ERP Analysis

The ERP waveforms were divided into a 50–ms time window from 100 (where T2 occurred) to 600 ms after T1 onset (10 time windows). The average amplitude within each time window was computed. This analysis method has been widely used to explore the time course of different ERP effects (Schirmer et al., 2005; Wu et al., 2017; Zou et al., 2019). Four representative regions of interest (ROIs), namely, left-anterior sites (F3, F5, FC3, and FC5), left-posterior sites (CP3, CP5, P3, and P5), right-anterior sites (F4, F6, FC4, and FC6), and right-posterior sites (CP4, CP6, P4, and P6), were calculated separately. The average amplitude values across the four representative scalp areas were calculated based on the combination of factors of hemisphere (LH and RH) and region (anterior and posterior sites). Grand averaged ERP waveforms were collapsed separately according to different visual field presentations and stimulus types. The representative electrode sites (CP3 and CP4) from the two ROIs (left posterior site and right posterior site) and scalp voltage maps of ERP transposition effect (transposed word data minus canonical word data) are plotted in **Figures 3**, **4**, respectively.

For each 50-ms time window, repeated measures analysis of variance (ANOVA) was performed with the stimulus type (canonical word vs. transposed word vs. pseudoword), visual field (LVF vs. CVF vs. RVF), hemisphere (LH vs. RH), and region (anterior site vs. posterior site) data. *P*-values were corrected by Greenhouse–Geisser correction when Mauchly's test of sphericity was violated (Greenhouse and Geisser, 1959), and the effect size partial eta-squared ($\eta_{p}^{2}$) was reported.

Summary results of the overall ANOVAs within each 50-ms time window are shown in Table 2, including the *F*-values of ANOVAs for the ERP waveforms involved in the stimulus type condition. As shown in **Figure 4**, there seem to be opposite patterns in character transposition effects between LVF and CVF, and between LVF and RVF. For significant interactions, further pairwise comparisons concentrated on the stimulus type effects for different visual field presentations separately. To protect against Type I error caused by further multiple comparisons, false discovery rate (FDR) correction (Benjamini and Hochberg, 1995) was applied with corrected *p*-values reported.

**Table 2:** *F*-values of the ANOVA for the ERP waveforms involved in the stimulus-type condition.

**Time windows (ms)**	**ST** **(*df* = 2.60)**	**ST × VF** **(*df* = 4.120)**	**ST × He** **(*df* = 2.60)**	**ST × Re** **(*df* = 2.60)**	**ST × VF × He** **(*df* = 4.120)**	**ST × VF × RE** **(*df* = 4.120)**	**ST × He × Re** **(*df* = 2.60)**	**ST × VF × He × Re** **(*df* = 4.120)**
100–150	1.22	0.58	0.07	0.67	0.86	1.58	0.76	1.21
150–200	1.44	0.43	2.93	0.13	0.77	0.64	2.39	2.27
200–250	1.78	1.08	1.39	0.89	0.36	0.85	0.73	1.17
250–300	1.91	4.58^**^	1.59	1.43	0.41	3.85^**^	3.09	0.82
300–350	4.71^*^	5.19^**^	2.90	3.05	1.05	4.46^**^	1.57	0.51
350–400	**10.09** ^***^	2.48^*^	3.72^*^	4.25^*^	2.61^*^	1.07	0.35	0.43
400–450	12.02^***^	3.59^**^	4.39^*^	0.74	2.95^*^	2.18	2.55	1.09
450–500	36.09^***^	8.04^***^	5.78^**^	0.76	1.96	2.08	0.53	0.26
500–550	60.82^***^	4.7^**^	3.78^*^	0.29	0.59	2.47	1.86	0.13
550–600	47.69^***^	2.86^*^	1.41	1.99	3.23^*^	1.15	4.92^*^	0.96

*Significant effects are shown by the bolded F-values*.

*ST, stimulus type; VF, visual field; He, hemisphere; Re, region*.

* *p < 0.05*;

** *p < 0.01*;

*** *p < 0.001*.

## Results

### Behavioral Results

**Figure 2:**
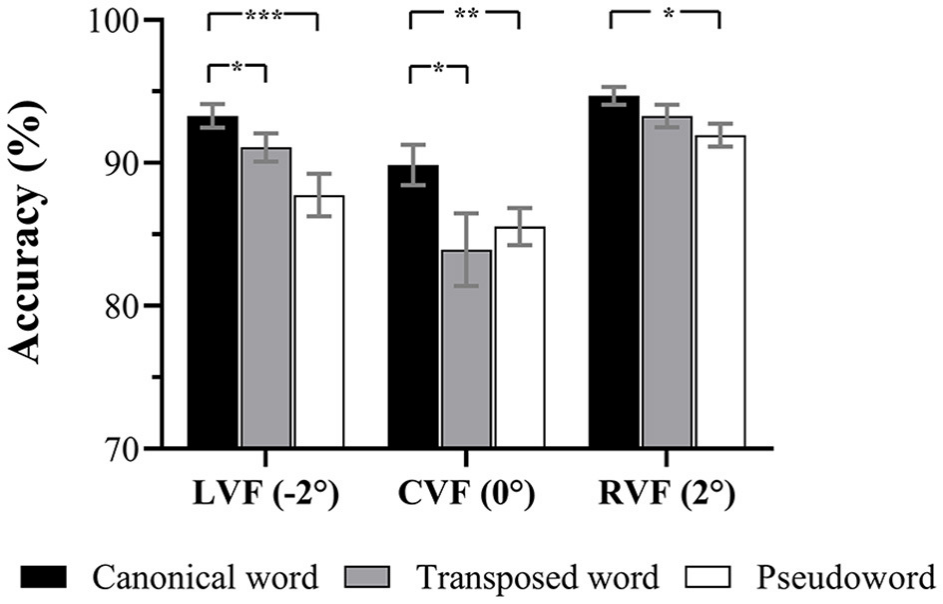
Mean accuracy of identifying T1 and T2 in the correct order. Error bars represent standard errors (SEs). ^*^p < 0.05; ^**^p < 0.01; ^***^p < 0.001.

First, the error rate of identifying T1 and T2 in a reverse order was typically higher in the CVF presentations (6.49% ± 6.11%) than in the LVF (1.58% ± 2.50%) and RVF (0.57% ± 0.99%) presentations, suggesting that order errors mainly occurred in the CVF presentations. Then, the mean accuracy of identifying T1 and T2 in correct order (see Figure 2) was analyzed *via* two-way repeated-measures ANOVA with visual field factors (LVF vs. CVF vs. RVF) and stimulus type (canonical word vs. transposed word vs. pseudoword). The main effects of stimulus type [F_(2,60)_ = 10.9, p < 0.001, $\eta_{p}^{2}$ = 0.27] and visual field [F_(2,60)_ = 16.99, *p* < 0.001, $\eta_{p}^{2}$ = 0.36] and the interaction effects [F_(4,120)_ = 3.89, *p* < 0.05, $\eta_{p}^{2}$ = 0.12] were all significant. Further pairwise comparisons with FDR correction showed that the accuracy for canonical words was significantly higher than that for pseudowords within each visual field (all *ps* < 0.05), indicating facilitation effects irrespective of the visual fields. There were also significant transposition costs in the form of lower accuracy for transposed words compared with that for canonical words with CVF presentations (*p* = 0.03) and LVF presentations (*p* = 0.03) but not with RVF presentations (*p* > 0.05).

When examining the visual field effects of each stimulus type separately, pairwise comparisons showed that there were no significant differences between the LVF and RVF in identification accuracies canonical words (*p* > 0.05) or transposed words (*p* > 0.05), except for pseudowords (*p* < 0.01), which showed an RVF advantage.

### ERP Results

There were no main effects of stimulus type or interaction effects in the first three 50-ms time windows (all Fs <2.93, *ps* > 0.05).

#### 250–300-ms Time Window

ANOVA revealed a significant three-way interaction effect between stimulus type, visual field, and region [F_(4,120)_ = 3.85, *p* < 0.01, $\eta_{p}^{2}$ = 0.11]. Further multiple comparisons showed that for LVF presentations, canonical words elicited more negative ERPs than transposed words at the anterior and posterior sites (both *ps* < 0.05), and pseudowords elicited a more negative ERP than transposed words at the anterior site (*p* < 0.05), but not at the posterior site (*p* > 0.05). No differences were found for CVF or RVF presentations (all *ps* > 0.05).

#### 300–350-ms Time Window

The three-way interaction effect between stimulus type, visual field, and region was significant [F_(4,120)_ = 4.46, *p* < 0.01, $\eta_{p}^{2}$ = 0.13]. Further multiple comparisons showed different patterns between LVF, CVF, and RVF presentations. For LVF presentations, canonical words elicited more negative ERPs than transposed words at the anterior and posterior sites (both *ps* < 0.05), and pseudowords elicited a more negative ERP than transposed words at the anterior site (*p* < 0.01) but not at the posterior site (*p* > 0.05). For CVF presentations, canonical words elicited less negative ERPs than transposed words and pseudowords at the anterior site (both *ps* < 0.01) but not at the posterior site (both *ps* > 0.05). No differences were found for RVF presentations (all *ps* > 0.05).

#### 350–400-ms Time Window

ANOVA revealed a marginally significant three-way interaction effect between stimulus type, visual field and hemisphere [F_(4,120)_ = 2.61, *p* = 0.04, $\eta_{p}^{2}$ = 0.08]. Further multiple comparisons found no differences for LVF presentations (all *ps* > 0.05). For CVF presentations, canonical words elicited less negative ERPs than transposed words and pseudowords in the LH and RH (all *ps* < 0.05). For RVF presentations, canonical words elicited a less negative ERP than pseudowords in the RH (*p* < 0.01) but not in the LH (*p* > 0.05), and no other differences were found (all *ps* > 0.05).

#### 400–450-ms Time Window

ANOVA also revealed a significant three-way interaction effect between stimulus type, visual field and hemisphere [F_(4,120)_ = 2.95, *p* < 0.05, $\eta_{p}^{2}$ = 0.09]. Further multiple comparisons found no significant differences for LVF presentations (all *ps* > 0.05). For CVF presentations, there were significant differences among canonical words, transposed words and pseudowords in the LH and RH (all *ps* < 0.05). For RVF presentations, canonical words elicited less negative ERPs than transposed words or pseudowords only in the RH (both *ps* < 0.05), but not in the LH (both *ps* > 0.05), and no other differences were found (all *ps* > 0.05).

#### 450–500-ms Time Window

ANOVA revealed a significant two-way interaction effect between stimulus type and visual field [F_(4,120)_ = 8.04, *p* < 0.001, $\eta_{p}^{2}$ = 0.21]. Further multiple comparisons showed significant differences among canonical words, transposed words, and pseudowords within each visual field presentation (all *ps* < 0.05). Specifically, canonical or transposed words elicited more positive ERPs than pseudowords regardless of the visual field. More importantly, for LVF presentations, transposed words elicited more positive ERPs than canonical words, but the opposite pattern was observed for CVF or RVF presentations.

#### 500–550-ms Time Window

ANOVA also revealed a significant two-way interaction effect between stimulus type and visual field [F_(4,120)_ = 4.7, *p* < 0.01, $\eta_{p}^{2}$ = 0.14]. Further multiple comparisons showed that canonical and transposed words elicited more positive ERPs than pseudowords within each visual field (all *ps* < 0.01). Moreover, there was no significant difference between canonical and transposed words for LVF presentations (*p* > 0.05), but canonical words elicited more positive ERPs than transposed words for CVF or RVF presentations (both *ps* < 0.001).

#### 550–600-ms Time Window

ANOVA revealed a significant three-way interaction effect between stimulus type, visual field, and hemisphere [F_(4,120)_ = 3.23, *p* < 0.05, $\eta_{p}^{2}$ = 0.1]. Further multiple comparisons showed that canonical words and transposed words elicited more positive ERPs than pseudowords in the LH and RH within each visual field (all *ps* < 0.05). Moreover, no difference was found between canonical words and transposed words for LVF presentations (both *ps* > 0.05), but canonical words elicited more positive ERPs than transposed words in the LH for CVF or RVF presentations (both *ps* < 0.01).

**Figure 3:**
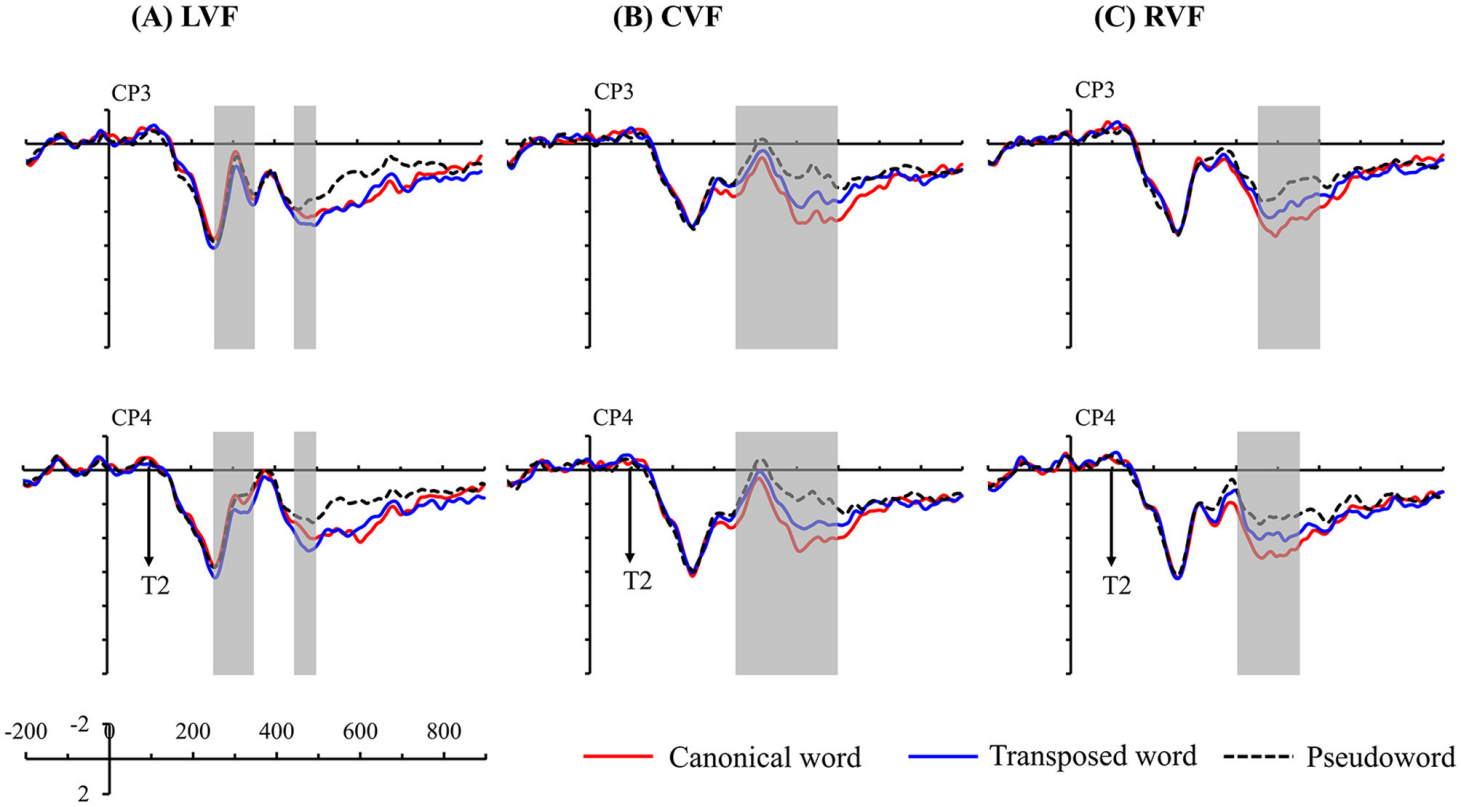
Average ERP waveforms in CP3 and CP4 under three conditions: **(A)** left visual field (LVF) presentation, **(B)** centrally presented visual field (CVF), and **(C)** right visual field (RVF) presentation. The gray rectangles indicate significant ERP differences between canonical words and transposed words at the left and right posterior sites.

In summary, canonical words elicited less negative ERPs than pseudowords in time windows 350–400 and 400–450 ms for CVF or RVF presentations, suggesting the occurrence of facilitation effects. The facilitation effects for both canonical words and transposed words were further observed during the latter 450–500, 500–550, and 550–600 ms time windows regardless of the visual fields. More importantly, in the earlier 250–300 and 300–350 ms time windows, the ANOVAs revealed less negative ERPs for transposed words than for canonical words for LVF presentations (see Figure 3A) but not for CVF or RVF presentations. However, in the subsequent five 50–ms time windows (350–400, 400–450, 450–500, 500–550, and 550–600 ms), significant transposition effects (more negative or less positive ERPs for transposed words than for canonical words) were found for CVF presentations (see Figure 3B). Moreover, in the latter 400–450, 450–500, 500–550, and 550–600 ms time windows, multiple comparisons also revealed similar significant transposition effects for RVF presentations (see Figure 3C). Notably, for RVF presentations, canonical words elicited less negative ERPs than transposed words or pseudowords in the RH but not in the LH in the time window of 400–450 ms.

## Discussion

In this study, we used ERPs with a dual-target RSVP task to explore the time course of neural dynamics involved in the processing of character transpositions in LVF, CVF, and RVF presentations. The behavioral results showed an RVF advantage for pseudowords but not for canonical or transposed words. This advantage might be modulated by the morphological combination of Chinese compound words. The accuracy for transposed words was significantly lower than that for canonical words in the LVF but not RVF presentations, indicating that combining the semantic information across two constituent characters of transposed words for LVF presentations required more extramental effort compared with RVF presentations. The electrophysiological results showed that transposed words elicited less negative waveforms than canonical words in time windows 250–300 and 300–350 ms in the LVF presentations but not in the CVF or RVF presentations. However, transposed words elicited more negative or less positive waveforms in time windows 400–450, 450–500, 500–550, and 550–600 ms than canonical words in the CVF and RVF presentations but not in the LVF presentations. These results indicated that visual field asymmetry and character transposition modulated the ERPs throughout the word processing stages, beginning at 250–300 ms and ending close to 550–600 ms.

The P200 time window is defined as ranging from 150 to 250 ms, which is usually thought to reflect the early phonological and/or orthographical processing of Chinese words (Kong et al., 2010, 2012; Zhou et al., 2014). Alternatively, the P200 component may also index the higher-level visual processing reported in prior studies that used lateralized visual field stimuli (Coulson et al., 2005; Wlotko and Federmeier, 2007). For instance, Coulson et al. (2005) found associative priming after the onset of targets for associated word pairs compared with unassociated word pairs in the P200 interval (150–250 ms) for both LVF and RVF presentations. In this study, however, neither the visual field asymmetries nor morpheme transpositions of Chinese compound words modulated the early P200 component.

**Figure 4:**
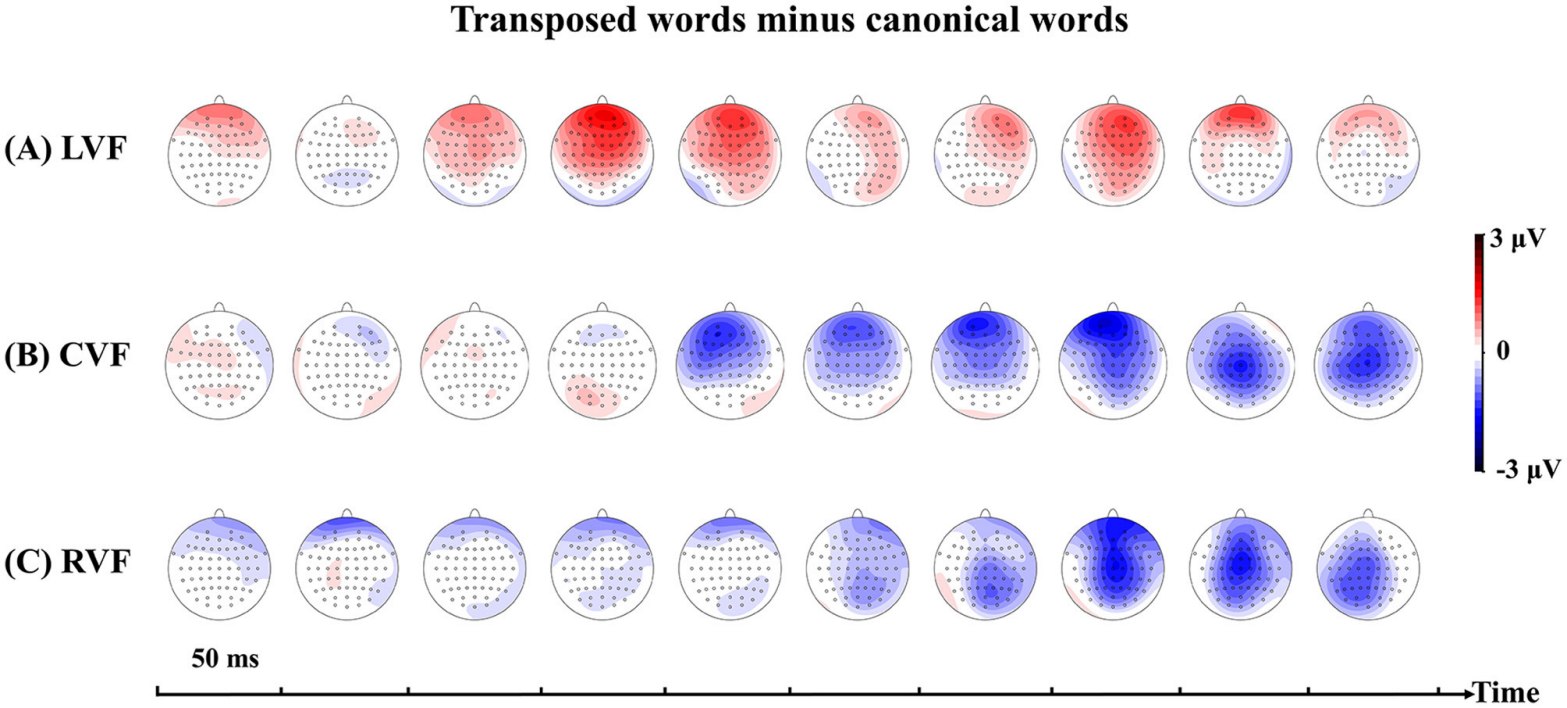
Scalp voltage maps of the ERP transposition effect (transposed word data minus canonical word data) for each analyzed 50–ms time window under three conditions: **(A)** left visual field (LVF) presentation, **(B)** centrally presented visual field (CVF), and **(C)** right visual field (RVF) presentation.

Furthermore, the comparative effects were the occurrence of two negative waves in the following four time windows (250–300, 300–350, 350–400, and 450–500 ms). These two negativities were labeled N250 and N400, respectively. Both components had a central-parietal scalp distribution and had been defined as brain potentials sensitive to form-level (Dunabeitia et al., 2009; Eddy et al., 2014) and semantic-level processing (Lau et al., 2009; Kutas and Federmeier, 2011; Lavric et al., 2011; Morris and Stockall, 2012; Leminen et al., 2019). Typically, the N250 component was in the time window of 200–300 ms and peaked at 250 ms (Grainger and Holcomb, 2009; Wu et al., 2020). However, the N250 component also started at ~240 ms, ended at approximately 350 ms, and peaked at ~300 ms (Okano et al., 2013). The N400 component could appear in the wide 200–600-ms time window, as noted by Kutas and Federmeier (2011). Researchers have demonstrated that the visual configuration of two-character Chinese words has its own contribution in triggering activation of the meaning of word (Ma et al., 2015). This study revealed a more negative N250 amplitude for canonical words than for transposed words, see Figures 3A, 4A, suggesting that transposed words could be more easily processed as “legitimate” compound words and further integrated into a whole for LVF presentations. Additionally, the relatively more positive waveforms elicited by the transposed words in the later 450–500 ms time window were also consistent with this deduction (see Figure 3A).

The N400 components were believed to reflect the retrieval of word meaning from semantic memory and the integration of the meanings of the constituent morphemes (Coch et al., 2013). The further striking findings of this study were the transposition effects, in the form of a more negative N400 amplitude for transposed words than for canonical words, which appeared only in the CVF and the RVF but not in the LVF, demonstrating the lexical/semantic access of transposed words required a more complex correction/suppression mechanism (Lo et al., 2019). The lack of N400 effects for LVF presentations might be modulated by the left-to-right scan habit. Note that from the scalp voltage maps in Figure 4, the transposition effect for CVF and RVF presentations mirrored the effect for LVF presentations, which also suggested that left-right asymmetries could affect the time course of lexical and semantic access during the character transposition processing of Chinese compound words. Previous studies have posited that the two targets were probably perceived as a single representation when they were presented successively during the RSVP task (Hommel and Akyurek, 2005; Dux et al., 2014). Following this logic, transposed words for LVF presentations were spatially real words, resulting in the comparable N400 effect compared with canonical words. In summary, these results suggested that visual field asymmetries and morpheme transposition could modulate not only form-level but also semantic-level representations of compound words.

Notably, the N400 facilitation effects for canonical words with RVF presentations were only apparent in the RH, which suggested that information had been shared between the two cerebral hemispheres *via* the corpus callosum by the time processing reached higher-level semantic processing (Brysbaert, 2004; Federmeier et al., 2010). Previous studies have used ERP measures with visual field presentations to examine differences in the process of each hemisphere to evaluate various semantic relationships, and have consequently proposed the coarse coding hypothesis; which suggested that semantic activation is fine-grained in the LH but coarse-grained in the RH (Jung-Beeman, 2005; Federmeier et al., 2010). The fine-coarse semantic coding theory claimed that the LH was involved in processing fine linguistic information, such as dominant word meaning, while the RH was responsible for processing coarse semantic information, such as unusual constructions (Beeman and Chiarello, 1998; Jung-Beeman, 2005). According to this theory, more involvement of RH would be expected for transposed words than for canonical words. The ERP findings were in favor of this hypothesis.

Finally, the LPC was the widespread positive-going wave in the final cluster (450–500, 500–550, 550–600 ms). The determination for this component was that the LPC component was typically within the 400–800 ms time window (Curran, 2000; Jang and Hyde, 2020), which varied with the processing manipulation and usually peaked after ~500 ms (Kim and Kim, 2006; Qiu et al., 2008; Beyersmanna et al., 2014). The LPC is usually thought to reflect more extensively explicit elaborate processing, such as semantic (Bouaffre and Faita-Ainseba, 2007; Tong et al., 2014; Zou et al., 2019), memory (Dunn, 1998; Evans and Federmeier, 2007; Strozak et al., 2016), and reconstruction (van de Meerendonk et al., 2011; Stites et al., 2016). As shown in Figures 3, 4, the typical LPC facilitation effects of canonical words were observed irrespective of the visual fields. This extensive LPC facilitation effect of canonical words for the three visual fields was consistent with the behavioral results, which suggested that the LPC in this study might reflect more explicit aspects of semantic processing and be related to a more general process of decision-making or successful comprehension. Finally, according to the split fovea model, position-specific encoding in the LH was more sensitive to transpositions for alphabetic languages (Shillcock and Monaghan, 2004). The ERP findings provided evidence to support the predictions of this model (Monaghan et al., 2004). This study revealed that transposed words elicited a more negative N400 amplitude and a less positive LPC amplitude compared with the amplitudes elicited by canonical words for CVF and RVF presentations.

## Conclusion

Taken together, although the RSVP paradigm with the visual field technique differs to some extent from normal reading, such experimental manipulations are sufficiently sensitive to detect the time course of word processing and hemispheric asymmetries. The results of this study indicated that both visual field asymmetries and morpheme transpositions of Chinese compound words could modulate the deflection of N250, N400, and the LPC. Specifically, character transposition facilitated the mapping of whole-word orthographic representation to semantic information in the LVF, as reflected by the less negative N250 component, and such morpheme position encoding influenced whole-word semantic processing in CVF and RVF presentations, as reflected by the more negative N400 and less positive LPC.

## Data Availability Statement

The raw data supporting the conclusions of this article will be made available by the authors, without undue reservation.

## Ethics Statement

The studies involving human participants were reviewed and approved by the Institutional Research Ethics Committee of Chongqing University. The patients/participants provided their written informed consent to participate in this study.

## Author Contributions

E-HZ and H-WC contributed to conception and design of the study and wrote the first draft of the manuscript. E-HZ and X-XL performed the experiments. E-HZ performed the statistical analysis. All authors contributed to manuscript revision, read, and approved the submitted version.

## Conflict of Interest

The authors declare that the research was conducted in the absence of any commercial or financial relationships that could be construed as a potential conflict of interest.

## Publisher's Note

All claims expressed in this article are solely those of the authors and do not necessarily represent those of their affiliated organizations, or those of the publisher, the editors and the reviewers. Any product that may be evaluated in this article, or claim that may be made by its manufacturer, is not guaranteed or endorsed by the publisher.
